# Supplementary material for: Modeling glioblastoma heterogeneity as a dynamic network of cell states
Source: Mol Syst Biol. 2021 Sep 16;17(9):e10105. doi: 10.15252/msb.202010105 (PMC8444284; doi:10.15252/msb.202010105)
Supplement: Supplementary file 6 — Source Data for Figure 5 [file MSB-17-e10105-s004.zip › Figure5A_sourcedata/GSEA_3017/nosigdb_stateE.GseaPreranked.1622795031093/index.html]

Index for xtools.gsea.GseaPreranked nosigdb\_state6.GseaPreranked.1622795031093

### GSEA Report for Dataset state63017

#### Enrichment in phenotype: **na**

- 78 / 139 gene sets are upregulated in phenotype **na\_pos**- 58 gene sets are significant at FDR < 25%- 51 gene sets are significantly enriched at nominal pvalue < 1%- 56 gene sets are significantly enriched at nominal pvalue < 5%- Snapshot of enrichment results- Detailed enrichment results in html format- Detailed enrichment results in excel format (tab delimited text)- Guide to interpret results

#### Enrichment in phenotype: **na**

- 61 / 139 gene sets are upregulated in phenotype **na\_neg**- 47 gene sets are significantly enriched at FDR < 25%- 42 gene sets are significantly enriched at nominal pvalue < 1%- 46 gene sets are significantly enriched at nominal pvalue < 5%- Snapshot of enrichment results- Detailed enrichment results in html format- Detailed enrichment results in excel format (tab delimited text)- Guide to interpret results

#### Dataset details

- The dataset has 947 features (genes)- No probe set => gene symbol collapsing was requested, so all 947 features were used

#### Gene set details

- Gene set size filters (min=15, max=500) resulted in filtering out 211 / 350 gene sets- The remaining 139 gene sets were used in the analysis- List of gene sets used and their sizes (restricted to features in the specified dataset)

#### Gene markers for the **na\_pos** *versus* **na\_neg** comparison

- The dataset has 947 features (genes)- Detailed rank ordered gene list for all features in the dataset

#### Global statistics and plots

- Plot of p-values *vs.* NES- Global ES histogram

#### Other

- Parameters used for this analysis

#### Comments

- Timestamp used as the random seed: 1622795031208

---

Report: nosigdb\_state6.GseaPreranked.1622795031093.rpt   by user: idala384

xtools.gsea.GseaPreranked [Fri, Jun 4, '21 10 AM 23]

Website: www.gsea-msigdb.org/gsea
Questions & Suggestions: Contact page
